# Using Aptamers for Cancer Biomarker Discovery

**DOI:** 10.1155/2013/817350

**Published:** 2013-01-15

**Authors:** Yun Min Chang, Michael J. Donovan, Weihong Tan

**Affiliations:** ^1^Center for Research at Bio/Nano Interface, Department of Chemistry and Department of Physiology and Functional Genomics, Shands Cancer Center, UF Genetics Institute and McKnight Brain Institute, University of Florida, Gainesville, FL, USA; ^2^Molecular Science and Biomedicine Laboratory, State Key Laboratory of Chemo/Bio-Sensing and Chemometrics, College of Biology, College of Chemistry and Chemical Engineering, Collaborative Innovation Center for Chemistry and Molecular Medicine, Hunan University, Changsha, 410082, China

## Abstract

Aptamers are single-stranded synthetic DNA- or RNA-based oligonucleotides that fold into various shapes to bind to a specific target, which includes proteins, metals, and molecules. Aptamers have high affinity and high specificity that are comparable to that of antibodies. They are obtained using iterative method, called (Systematic Evolution of Ligands by Exponential Enrichment) SELEX and cell-based SELEX (cell-SELEX). Aptamers can be paired with recent advances in nanotechnology, microarray, microfluidics, and other technologies for applications in clinical medicine. One particular area that aptamers can shed a light on is biomarker discovery. Biomarkers are important in diagnosis and treatment of cancer. In this paper, we will describe ways in which aptamers can be used to discover biomarkers for cancer diagnosis and therapeutics.

## 1. Introduction

Approximately 1.5 million Americans were diagnosed with cancer in 2010 [1]. Malignant neoplasm is the second leading cause of death in the world and the leading cause of death in developed nations [2]. Chemotherapy is a common method of treating cancer, but it is largely indiscriminate in that it does not target cancer cells with specificity. Therefore, considerable interest has been shown in developing novel treatments that target only cancer cells, thus avoiding the toxicity of chemotherapy against normal tissues adjacent to the tumor. Such targets can be cancer-specific biomarkers that may be used to assess the changes in expression states of certain proteins or genes within a primary tumor. Since genetic mutations play a key role in modulating the maintenance and progression of cancer cells, fundamental differences in protein levels or gene expression states can be exploited and used for diagnostics and therapies [3]. This paper aims to shed light on the possibility of utilizing aptamers for the discovery of crucial biomarkers for cancers with the goal of improving early-stage diagnosis and therapy.

In recent years, interest has been shown in using aptamers to develop cancer treatments. Currently, AS1411 [4, 5], a potential therapeutic for acute myeloid leukaemia, and NOX-A12 [6, 7], a potential therapeutic for multiple myeloma and non-Hodgkin's lymphoma, aptamers developed by Antisoma and NOXXON, respectively, are in clinical trials [8]. Aptamers are single-stranded oligonucleotides that act like antibodies in recognizing molecular moieties like biomarkers [9]. Because of their ability to fold into secondary or tertiary shapes, aptamers can bind to a wide range of targets, such as metals, proteins, biological cells and tissues, with high specificity [10]. Aptamers generated against various cancer cell lines can be used initially for biomarker discovery and later for diagnostic and therapeutic purposes.

## 2. Aptamers for Biomarker Discovery

Biomarkers can be expressed in different forms, including, for example, proteins unique to cancer types and subtypes [11]. Proteins are the most useful form of biomarkers since they mirror the genotype and phenotype of a particular disease. Furthermore, since proteins reflect a cell's phenotype, physical alterations of proteins within the cell, as well as overexpression and downregulation of certain proteins, can have profound effects on the cell as a whole. This alteration in protein composition can be the result of posttranslational modification [12] or mutation at the genetic level, as in the case of cancer [13]. However, it is challenging to develop a biomarker system able to provide accurate evidence that a protein or oncogene is a reflection of a cell's physiological state at some defined stage, essentially because of the robust procedures required to screen for such biomarkers at specific stages of carcinogenesis. These stages of development may not simply lead to overexpression of a single protein but may lead to a change in the ratio of certain proteins. In this case, merely identifying the presence of a protein is not satisfactory. A detection platform that is able to quantify the expression levels of a variety of protein will deliver valuable information. Multiplexed systems that incorporate aptamers for a variety of proteins can be performed, and the aptamers that do bind to protein can be quantified via microarray analysis in order to determine the ratio of specific proteins.

**Figure 1:**
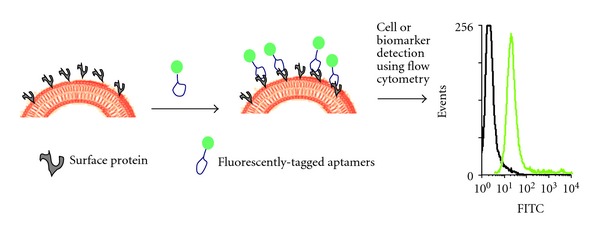
Detection of surface proteins or biomarker in cancer cells using fluorescently tagged aptamers.

Thus far, the attempts to screen biomarkers have only seen modest results. One traditional method has been the incorporation of antibodies. Yet, antibodies often require a sandwich system in order to detect their target protein [14]. This means that two different types of antibodies must be able to properly identify the target, making large-scale biomarker screening impractical under these conditions. Aptamers, on the other hand, have the ability to overcome such pitfalls, making protein biomarker applications suitable in clinical settings (Figure 1). Aptamers are target specific, able to penetrate cellular membranes, and they can be inexpensively modified and synthesized.

**Figure 2:**
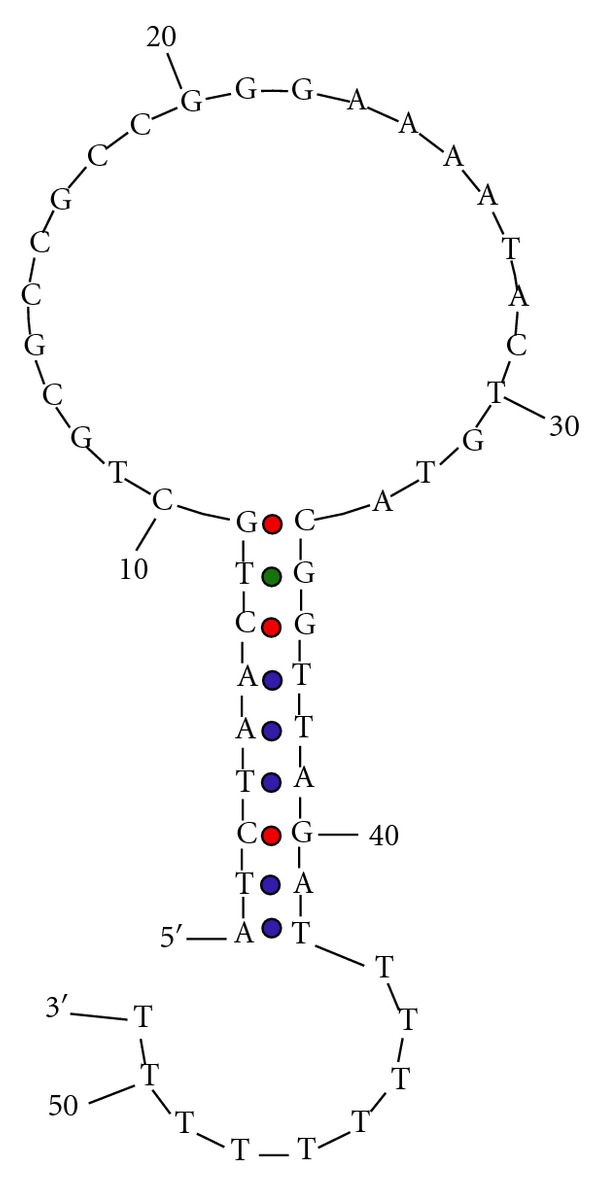
Predicted folded structure of sgc-8 aptamer generated by mfold [17].

A multitude of applications are available for aptamers, including drug delivery, molecular imaging, and diagnostics. Above all else, however, aptamers for use in biomarker discovery respond to a critical need. Specifically, aptamers can distinguish among thousands of proteins and do so in a short period of time, and they can detect small differences between proteins that are otherwise quite similar in structure, an essential property if proteins are to be differentiated on the cell surface.

(Systematic Evolution of Ligands by Exponential Enrichment) SELEX and cell-based SELEX (cell-SELEX) [15] are two *in vitro* methods used to generate aptamers by iterative positive and negative selection processes that ultimately eliminate non- or weak binding candidate sequences. Aptamers selected in this manner can target overexpressed proteins on the cell surface and even detect small differences among cell-surface proteins. This capability allows aptamers to differentiate unique cellular characteristics, particularly those between cancerous and noncancerous cells based on their apparent biomarker or unique cell-surface homology. Such strategy was used by our group to identify a biomarker for T-cell acute lymphoblastic leukemia (T-ALL), the transmembrane protein tyrosine kinase 7 (PTK7) [16]. By using cell-SELEX, a method akin to SELEX, but one in which live cells are utilized for unbiased selection of aptamers that bind to native forms of the proteins or target, present aptamer sgc8 was selected to bind tightly to PTK7 (*K*_*d*_ = 0.80 ± 0.09 nM) [17] (Figure 2). PTK7 was subsequently identified as a potential biomarker for T-ALL. Furthermore, while sgc8 displayed high selectivity and affinity for its target on most T-ALL and acute myeloid leukemia (AML) cells, as well as some B-cell acute lymphoblastic leukemia (B-ALL) cells, its detection levels in lymphoma and normal human bone marrow cells were not comparable [17] (Table 1). This implies that PTK7 plays a role in the development of most T-ALL and AML cases. Thus, the fundamental differences in expression levels of surface proteins, elucidated with the help of aptamers, play a key role in our ability to differentiate between normal and cancerous cells. 

Another example of biomarker discovery using cell-SELEX is the discovery of tenascin-C aptamers using glioblastoma cell line, U251 [18]. This *a priori *approach to cell-SELEX helped to reveal biologically pertinent proteins that play a role in cancer that were not previously known. The authors attribute the selection of tenascin-C aptamers using U251 cell lines to the abundance and accessibility of tenascin-C within glioblastoma. This inferred knowledge of overabundance of tenascin in tumor cells was not a coincidence. Tenascin protein was first demonstrated in 1989 as an extracellular matrix protein present in the fetal rat mesenchyme around mammary glands, hair follicles, and teeth, where developmental mesenchyme is present and necessary [19]. Interestingly, adult mammary glands did not contain tenascin, however, when the mammary glands were exposed to carcinogens producing induced tumors, tenascin levels were detectable in fibrous tissues [19]. Upon further inspection, it was shown that tenascin could play a vital role in and serve as a stromal marker for epithelial malignancies. Yoshida et al. report that tenascin is secreted by human lung cancer and glioma cells at a concentration ranging from 1 to 14 *μ*g/mL *in vitro *[20]. Further, patient samples of cerebrospinal fluid with brain tumors showed an increase of tenascin present by >100 ng/mL [20]. Later, immunohistochemical assays revealed that 50 sections of human ovarian tumors showed a greater intensity of staining of tenascin proteins correlating to the high expression of tenascin in cancer cells [20]. Furthermore, malignant and borderline tumors had higher expression levels of tenascin compared to benign tumors [21]. These previous reports of tenascin's role in cancer confirm the aptamer's ability to elucidate how expression levels of certain oncogenic proteins can be used to differentiate between cancerous and noncancerous cells.

Aptamers can not only provide a way of discovering biomarkers that help to differentiate between cancer cells and normal cells, but they can also help to differentiate the stage of carcinogenesis within one subtype of cancer. One study using a DNA-based aptamer with very specific affinity for protein binding revealed 44 different biomarkers able to distinguish among Stage I–III lung cancer [22]. Furthermore, each biomarker correlated with such cofactors as age and frequency of smoking for better treatment outcomes [22]. Under these conditions, early cancer detection is possible, and aptamer-based biomarker discovery with “staging” capability makes it further possible to assess the progression of cancer in terms of the best treatment options.

**Figure 3:**
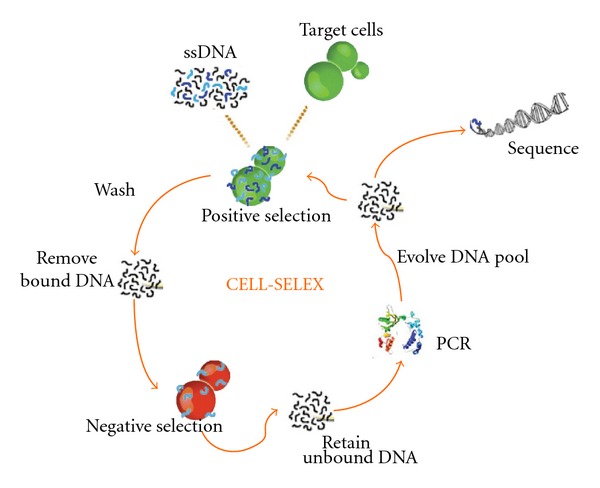
Schematic diagram of cell-SELEX [10].

Unlike SELEX, cell-SELEX uses whole living cells to avoid the disadvantage of selecting aptamers against a nonnative protein conformation (Figure 3). As previously mentioned, this strategy does not require any prior knowledge of the target proteins' conformations or their expression levels within the cell. Since aptamers have the potential of being used in living systems, especially within human biological condition, selecting aptamers using proteins in their native conformation and conditions alleviates the possibility of selecting physiologically nonfunctional aptamers. Furthermore, it bypasses the need for target protein purification, a process that may further disrupt the native conformation of the protein. Cell-SELEX allows all cell-surface molecules or protein to be retained in their native environment, thereby retaining their native folding and physical structures including possible posttranslational modifications throughout the selection process. Thus, aptamers selected with the use of whole live cells will bind to the target with appropriate modifications and conformation on cells. The ability of selecting aptamers in this manner shows great potential in biomedical research and in the development of cell-specific diagnosis and therapeutics in physiological conditions. In addition, using cell-SELEX provides us with the advantage of an unbiased approach to discovering biomarkers for various diseases, leading to improved cancer diagnostics and therapeutics. We say “unbiased” because prior knowledge of a potential biomarker is not necessary to discover a biomarker using a cell-SELEX approach. “Biased” approach to aptamer selection would mean that a particular biomarker for a certain cancer is already known, but an “unbiased” approach would lend a method of contributing to the discovery of the biomarker itself. This inherent advantage of this approach is the targeting of a disease state, such as cancer, without prior knowledge of the disease's molecular differences. Tumor cell-SELEX provides us with the ability of discovering known and unknown tumor biomarkers [23]. This strategy has the potential of allowing us to discover other uncharacterized biomarkers that illuminate our knowledge on tumor presence, genesis, and progression. It is also likely that discovery of other biologically interesting targets, not previously correlated or connected with cancer, is a possibility. Once such aptamer is selected, it can not only advance diagnosis and treatment of cancer but also help to reveal important biology that defines cancers.

Theoretically, we should be able to use cell-SELEX to profile large numbers of primary tumor cell lines and discover biomarkers for each cancer. Furthermore, such biomarker discoveries can parallel existing cancer genomic data to generate connection between protein biomarkers and genomic biomarkers that differentiate cancerous and non-cancerous cells. This knowledge can provide researchers to develop targeted therapies for cancer that minimize killing of normal cells and physicians a way to treat cancer based on molecular information as opposed to preexisting morphological information of tumors.

## 3. Imaging Cancer Cells Using Biomarkers and “Membranome”

The term membranome refers to a set of biological membranes that exist in a specific organism. The term was coined by a British biologist, Cavalier-Smith [24], in a way to describe the epigenetics of biological membranes. The term has also been applied to an entire set of proteins [25] or a combination of membrane proteome and lipidome. It is this set of proteins on the cell surface membrane that constitutes its membranome and is a key to understanding biomarker discovery by using aptamers. As previously mentioned, aptamers can be used to detect and differentiate among various cancer cells and their noncancerous subtypes. Such detection is possible because the binding of aptamers occurs mainly on the extracellular domains of cell-surface proteins [26]. This ability of aptamers to bind to such proteins provides a way to identify and discover proteins characteristic of certain cancers. By first characterizing a common protein expressed in a certain cancer, it is possible to image cancer cells by tagging fluorescent dyes to the aptamer's tail. The dyes provide a way to see cancer cells with the naked eye (Figure 1).

Imaging of cancer cells can be enhanced by coupling nanoparticles to aptamers. For example, nanoparticles (NPs) can be bioconjugated with aptamers and used in a colorimetric system where color change occurs upon particle aggregation [10]. In another instance, a multimodal nanoparticle was conjugated with aptamer AS1411 to detect nucleolin, a protein commonly expressed on the membrane of cancer cells, making it possible to image and detect the presence of cancer [9]. By using fluorescence, radioisotope, and MRI imaging techniques, it is possible to detect cancer cells *in vivo* and *in vitro* [9]. Although NPs are toxic *in natura*, by surrounding them with a stable silica shell, or surface modification using aptamers, it might be possible to eliminate the risk of toxicity and conduct clinical studies [9]. This capability also provides a new and improved method to image cancer cells during surgery or develop nanomedicine treatments.

## 4. Detection of Rare Cancer Cells Using Aptamers

**Figure 4:**
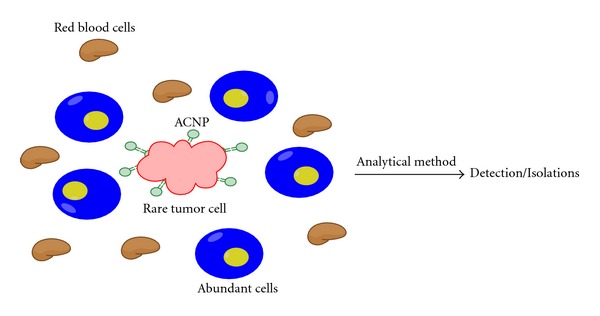
Biomarker concentration within a matrix can be very low. By using aptamer-conjugated nanoparticles, it is possible to detect and discover trace amounts of biomarkers or cells [30].

Interestingly, in cancer, circulating tumor cells (CTC) [27, 28] and secreted cancer biomarker, peptide growth factors, cytokines, and hormones [29] can be used as diagnostic markers for cancer diagnosis. Unfortunately, the number of CTCs and other rare secreted protein biomarkers is few. However, we can use aptamers to alleviate detection complications, which can lead to innovations in early cancer detection. This detection method involves testing bodily fluids, such as blood, serum, and sputum, where malignant tumors are present but in very low abundance relative to the large concentration of background cells [10]. To solve this problem, our group has developed aptamer-conjugated nanoparticles (ACNPs) to detect and extract cancer cells from matrices like blood and serum [1], surpassing the capability of antibody-based biomarker discovery. The working principle of ACNPs is conjugating highly selective aptamers to silica-coated magnetic and fluorophore-doped silica nanoparticles to detect and extract target cells from various matrices. Accordingly, ACNPs have shown a theoretical limit of detection (LOD) of 6.6 cells within a complex matrix which is the lowest LOD measured compared to the single-aptamer NPs at 152 cells and original assay LOD of 250 CEM cells [30] (Figure 4). The limit of detection (LOD) was calculated by adding the blank standard deviation multiplied by 3 to the blank signal and determining the LOD from the equation of the line generated by Microsoft Excel. ACNPs are applicable to many types of cancer cells because adaptations, such as chemically modifying aptamers, conjugating different targeted aptamers or changing the type of nanoparticles, can be made for the imaging, detection, and extraction of various cancer cell lines [30]. The versatility and the sensitivity of ACNPs can improve CTC and other rare biomarkers released by cancers detection to change how we diagnose cancer. Such isolation and separation of cancer cells can further be enhanced using aptamer-based fluorescence-activated cell-sorting device as reported by the Famulok group [31]. This method improves cell sorting by reducing false-positive detection of cells that are bound to aptamers due to cellular death or other membrane complications. This approach can be paired with the use of ACNP to accurately detect biomarkers and cancer cells in low concentrations.

## 5. Cell Profiling Using Aptamers and Biomarkers

After cancer cells have been isolated from complex media, aptamer-based cell profiling allows for differentiation between cancer and normal cells based on different protein expression levels. Fluorescent aptamers can be used to profile and study many different types of cancer cells.

**Figure 5:**
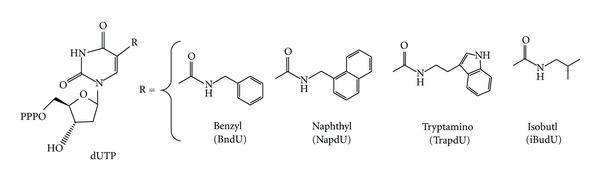
Modifications made for SOMAmer. The nucleotide triphosphate analogs were modified at the 5-UTR position (R) of uridine (dUTP), 5-benzylaminocarbonyl-dU(BndU); 5-naphthylmethylaminocarbonyl-dU (NapdU), 5-tryptaminocarbonyl-dU (TrpdU), and 5-isobutylaminocarbonyl-dU (iBudU) [8].

The George Church's group at Harvard Medical School [32] has recently developed a logic-gated nanorobot using aptamers for targeted payload delivery. They used aptamer sgc8, which was positively selected using acute lymphoblastic leukemia cells (CCRF-CEM) and negatively selected against a Burkitt's lymphoma cell line (Ramos) [33]. Sgc8 specifically binds PTK7, a receptor present on CCRF-CEM cells, but not Ramos cells. Sgc8-gated nanorobots, which specifically bind and open only in the presence of PTK7 proteins, can recognize the expression level of PTK7 in various cell lines [33]. In another example, Gold et al. have demonstrated the ability of their slow off-rate modified aptamers (SOMAmer) to accurately and specifically recognize cell-surface proteins. Their aptamers modified the uridine 5′-triphosphate (UTP), as illustrated in Figure 5.

Gold et al. further used the modified SOMAmers to develop aptamers for 813 human proteins. Prior to modification, the success rate for protein targeting was <30%. After modifications incorporating the four nucleotides, as shown in Figure 5, the success rate for obtaining an aptamer with a *K*_*d*_ less than ~30 nM was approximately 84% [14]. This is a tremendous increase and one that allows aptamers to be viable components in applications involving biomarkers.

For multiplexed system that Gold et al. incorporated, the reactions took place in a solution, not on a surface. This allowed for the advantageous use of kinetics. In solutions where aptamers with a higher binding affinity are present, they will act as a competitive inhibitor against aptamers with lower binding affinities. Aptamers that did not bind to any proteins were washed away while the ones that did bind to proteins present were kept to be analyzed. A microarray of complementary strands to the aptamers was prepared. This microarray was able to quantify the aptamers present after washing, effectively giving a quantity for the protein levels present. This multiplexed system not only was able to analyze a large array of aptamers but also able to collect data to determine the significance of certain ratios of proteins present.

## 6. Aptamers for Known Biomarkers

Validating biomarkers is a challenging exercise; accordingly, few *bona fide* proteins have been identified. However, one glycoprotein has been thoroughly studied and generally agreed to be a biomarker for prostate cancer. This 33-34 kDa glycoprotein is termed prostate specific antigen (PSA). Serum PSA is released into the blood stream after being produced by the prostate epithelium. By using the SELEX method and using a unique genetic algorithm, Ikebukuro et al. were able to derive an aptamer sequenced for PSA. The genetic algorithm was incorporated after traditional SELEX methods. The aptamers obtained had a *K*_*d*_ in the tens of nanomolar range. The group was also able to sense PSA concentrations between 40 and 100 nM when incorporating their aptamers [33]. Such aptamer construct can be used for biosensing.

The finding by Ikebukuro et al. also raises an important point as to the proximity of biomarkers. Antigens, which are present in the blood, may signal a certain disease is present, but do not give evidence to its exact location. For biomarkers that are closely positioned to the cancerous cells, aptamers may be used in conjunction with drugs in order to develop a theranostic platform. Yet, if the biomarker is not in direct proximity to the cancer cell, an additional form of testing will need to be administered to determine where the unhealthy cells are. It is clear that not all biomarkers are the same in terms of their applications. For biomarkers such as PSA, their use is strictly diagnostic. For biomarkers that are on the unhealthy cells surface, they can serve in diagnostics and also be used as a target for therapy.

## 7. Conclusions

As indicated in this paper, aptamers have shown promise in cancer studies, clinical diagnostics and therapeutic applications. Importantly, aptamers can be used for both clinical purposes, as well as biomarker discovery. However, to further advance the use of aptamers in the laboratory and clinical settings, the number of aptamers for specific targets must be increased. Also, there should be a push to select for aptamers that bind to cancer-specific or other disease-specific proteins that are outlined by cancer genomic studies and resources such as (Catalog of Somatic Mutations in Cancer) COSMIC [34, 35], OncoMap [36], and (The Cancer Genome Atlas) TCGA [37–42]. Using the cell-SELEX technique, previously unknown overexpressed proteins on the surface membrane of cancer cells can be identified as potential markers of carcinogenesis. At the same time, however, cell-SELEX relies on saturation levels of surface proteins, and it can be difficult to discover rare biomarkers that have low abundance on the cell membrane. Fortunately, negative selection for surface proteins known to be overabundant within the cell can circumvent this problem. Thus, aptamers can provide a systematic and accurate way to discover biomarkers for cancer, as well as other diseases, and a theranostic platform for practical clinical applications, particularly early-stage cancer detection.

## Figures and Tables

**Table 1:** Specificity of sgc8 aptamer for T-ALL cancer cells. binding capacity of the aptamer to the cells. 0: <10%; +: 10%–35%; ++: 35%–60%; +++: 60%–85%; ++++: >85%; APL: acute promyelocytic leukemia [17].

Cultured cell lines	sgc8
Molt-4 (T cell ALL)	++++
Sup-T1 (T cell ALL)	++++
Jurkat (T cell ALL)	++++
Sup-B15 (B cell ALL)	+
U266 (B cell myeloma)	0
Toledo (B cell lymphoma)	0
MO2058 (B cell lymphoma)	0
NB-4 (AML, APL)	0
